# How are pay-for-performance schemes in healthcare designed in low- and middle-income countries? Typology and systematic literature review

**DOI:** 10.1186/s12913-020-05075-y

**Published:** 2020-04-07

**Authors:** Roxanne J. Kovacs, Timothy Powell-Jackson, Søren R. Kristensen, Neha Singh, Josephine Borghi

**Affiliations:** 1 London School of Hygiene and Tropical Medicine, Faculty of Public Health and Policy, London, UK; 2 Imperial College London, Faculty of Medicine, Institute of Global Health Innovation, London, UK

**Keywords:** Pay-for-performance, Performance-based financing, Financial incentives, Health financing, Incentive design, Low and middle-income countries

## Abstract

**Background:**

Pay for performance (P4P) schemes provide financial incentives to health workers or facilities based on the achievement of pre-specified performance targets and have been widely implemented in health systems across low and middle-income countries (LMICs). The growing evidence base on P4P highlights that (i) there is substantial variation in the effect of P4P schemes on outcomes and (ii) there appears to be heterogeneity in incentive design. Even though scheme design is likely a key determinant of scheme effectiveness, we currently lack systematic evidence on how P4P schemes are designed in LMICs.

**Methods:**

We develop a typology to classify the design of P4P schemes in LMICs, which highlights different design features that are a priori likely to affect the behaviour of incentivised actors. We then use results from a systematic literature review to classify and describe the design of P4P schemes that have been evaluated in LMICs. To capture academic publications, Medline, Embase, and EconLit databases were searched. To include relevant grey literature, Google Scholar, Emerald Insight, and websites of the World Bank, WHO, Cordaid, Norad, DfID, USAID and PEPFAR were searched.

**Results:**

We identify 41 different P4P schemes implemented in 29 LMICs. We find that there is substantial heterogeneity in the design of P4P schemes in LMICs and pinpoint precisely how scheme design varies across settings. Our results also highlight that incentive design is not adequately being reported on in the literature – with many studies failing to report key design features.

**Conclusions:**

We encourage authors to make a greater effort to report information on P4P scheme design in the future and suggest using the typology laid out in this paper as a starting point.

## Introduction

Pay for performance (P4P) schemes – also called performance-based financing or results-based financing schemes – provide financial incentives to health workers or facilities based on the achievement of pre-specified performance targets and have been implemented in health systems across low and middle-income countries (LMICs). Numerous studies have been conducted on the effects of these schemes in LMICs, the results of which are summarised and discussed in a number of reviews [1–8]. Two issues become apparent when examining this evidence base. First, there is substantial variation in the effect of P4P schemes on a range of important outcomes, such as maternal and child health, utilisation of healthcare services or provider motivation. Furthermore, P4P schemes can have positive effects in some settings [9] – but can have no detectable effects on important targeted outcomes [10, 11], or even negative effects [12] in other settings. Second, a review of previous studies indicates that P4P is not a uniform intervention [5]. The large number of P4P programmes implemented in LMICs has brought with it a range of P4P designs – e.g. paying healthcare providers between one monthly salary per quarter [13] to no financial bonus at all [14] or incentivising outcomes as different as child nutritional status [15] and content of care during ante-natal care visits [16].^1^ When these two ideas are taken together, this leaves open the possibility that the heterogeneous results we observe relating to the impact of P4P schemes in LMICs could be (at least partially) attributed to differences in scheme design. Hence, it is possible that, ceteris paribus, some P4P designs work well whilst others do not.

Despite the a priori importance of P4P scheme design on outcomes, studies that conceptualise and summarise the design of P4P schemes have focused on high-income settings [17–25]. We therefore lack systematic evidence on how P4P schemes are designed in LMICs. However, given the difference in health system context between many LMICs and high-income countries, and the different reasons for introducing P4P in these settings, there is a need to develop a typology that focuses on the design of P4P schemes in LMICs. This study begins to fill this gap in the literature. This paper aims to first develop a typology of P4P designs in LMIC, and then aims to use this typology to classify and describe the design of P4P schemes that have been evaluated in LMICs in the academic and grey literature. In so doing, we reflect on whether incentive design has adequately been reported on and we make recommendations for reporting in future studies.

## Methods

### Developing a typology of P4P scheme design

We developed a typology to highlight design features of P4P schemes that are likely to affect the behaviour of those incentivised in relation to meeting incentivised targets. As our focus was on scheme design; we did not consider operational questions, such as which types of designs are easier or cheaper to implement.

To develop the typology, we followed three main steps. First we identified potentially relevant design features through: deliberation among the authors, who work on P4P in LMICs and high income countries, drawing on their knowledge of the design of P4P schemes; a review of studies that provide conceptual frameworks relating to the design of P4P schemes, in particular [17–24]; and a review of classical and behavioural economic theories on the relationship between incentive design and behaviour. Second, we created an initial draft of the typology including the design features initially identified. Third, we refined the typology by reducing the number of features by merging those that capture similar or the same underlying idea, and while populating the typology with data from studies identified in the review.

**Fig. 1:**
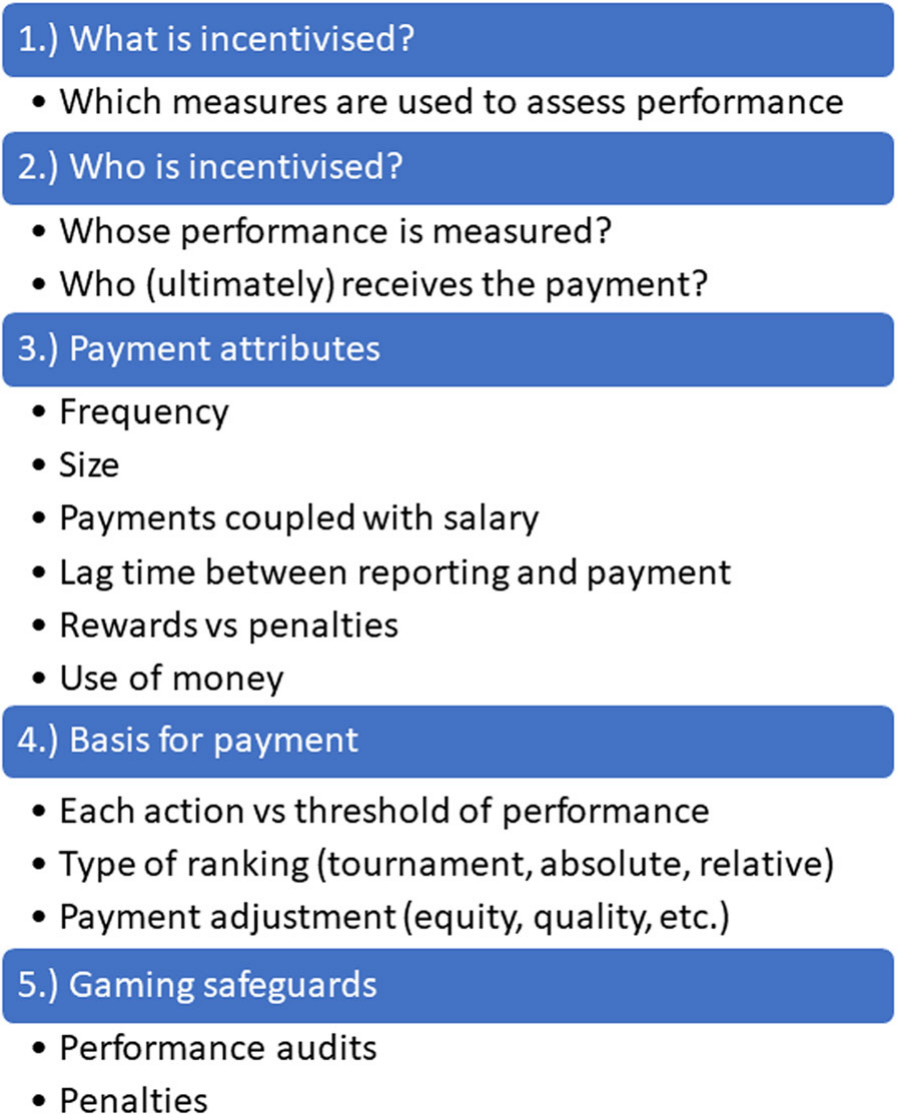
Typology for the design of P4P schemes in LMICs

Following the process described above, we identified five main design features, as shown in Fig. 1. The first design feature captures *what* is incentivised – meaning, which types of performance measures are rewarded in the P4P scheme. P4P schemes in the health care setting typically incentivise six types of measures: healthcare visits (consultation or service volumes), efficiency (average length of stay, patients seen per provider), structural quality of care (infrastructure, equipment, drug availability in the facility), process quality of care (content of care during medical consultations, e.g. whether iron tablets were given during antenatal care or patient satisfaction), management practices (supervision, use of medical records) or health outcomes (e.g. child nutritional status or anaemia levels). The main reason why the *type* of performance measure that is being incentivised is likely to be consequential for behaviour, is that providers have different degrees of control over different types of outcomes, and therefore have varying ability to respond to incentives [25]. For instance, providers have far less control over health outcomes, than they do over indicators that capture process quality of care. Moreover, some performance measures are more prescriptive in terms of the behaviours needed to achieve targets. For instance, schemes that incentivise process quality of care will much more clearly identify the behaviours needed to achieve a target, than those incentivising health outcomes.

The second design feature captures who is incentivised. We separate this out into two elements: whose performance is measured and who (ultimately) receives the payment. This can be individuals (e.g. individual doctors), groups (e.g. groups of providers working in the same healthcare facility, such as those working in the same department of a hospital) or institutions (e.g. healthcare facilities). Schemes that target groups are more likely to catalyse changes in group culture – which can lead to socialization or better selection of members, collaboration and peer-pressure to achieve results [26] – whilst schemes that target individuals are arguably better at preventing free-riding [27]. This design feature also captures whether or not P4P schemes measure the performance of and/or reward the performance of managers as this might create additional managerial pressure for performance.

The third design feature captures the attributes of the incentive payment. We include six characteristics of incentives that are likely to influence provider behaviour [28]: frequency of incentive payment, size of incentive, whether bonus and salary payments are coupled, lag time between performance reporting and when the incentive payment is made, whether incentives are rewards or penalties, and how the money can be used. The frequency and size of the payment determine the amount of the reward in a specific time period. Mental accounting theory [29] predicts that individuals would value bonus payments more highly if they are not coupled with their usual salary – since bonus payments are then likely to be considered a separate and additional income category. Principal agent theory suggests that incentive size will have a positive effect on performance, with diminishing marginal returns [30, 31]. The delay between taking an action and receiving payments is a priori relevant as a design feature because people more strongly discount benefits that occur in the future, therefore – the longer the delay the smaller the incentive [32]. Furthermore, particularly in settings where there is limited trust in the health system, long delays between reporting and payment could make individuals question whether incentives will be paid at all. Whether schemes provide rewards or penalties is arguably important because individuals experience losses and gains differently. As described in [25], this is due to loss aversion, as individuals feel losses more strongly than gains [33]. However, schemes that provide penalties might have a negative effect on morale, which could counteract the loss-aversion prediction [25]. Finally, we highlight how money gained through P4P schemes can be used. Traditionally in LMICs, additional funding can either be spent on staff bonuses or facility improvements (purchasing drugs, equipment or investing in facility renovation) or both. Different attributes of incentives are likely to have differential effects on providers’ working environment (and potentially their ability to reach targets) or increase the income of workers.

The fourth design feature is the basis for payment, which essentially captures the calculation or formula used to determine the size of the incentive payment. We highlight three main elements of this calculation: whether schemes reward each action or performance above a threshold, the type of ranking used and whether the payment is adjusted for factors like equity or quality of care. Whether schemes reward each action (for example, each woman who is given tetanus toxoid during an ante-natal care visit) or a threshold target (50% of women are given tetanus toxoid during ante-natal care visits) is a priori important for provider behaviour. On the one hand, schemes that reward each action are associated with less uncertainty than those rewarding a performance threshold – since providers know that each action is associated with a certain reward. On the other hand, schemes that use thresholds might induce a goal gradient response – where individuals become highly motivated when they are close to achieving a goal they think can be attained and less motivated otherwise (i.e. when they are far off or above the threshold) [34]. The type of ranking is also likely to affect the degree to which providers respond to incentives.

We distinguish between schemes that use a tournament design (that only rewards the best performing providers, such that providers are competing against one another), those that reward providers’ own *absolute* performance and those that reward providers for *improvements* in their own performance. These designs differ in two main respects. First, they are associated with different levels of uncertainty. For instance, in a tournament design it is unclear to providers whether any rewards will be received (since payoffs depend on the performance of others), which is not the case in schemes that use an absolute or relative performance threshold. Second, these designs differ in the degree to which they create “losers” and trigger loss-aversion. Unlike schemes that reward absolute or relative performance, a tournament design creates clear winners and losers, which might trigger loss-aversion and arguably improve performance. Finally, whether P4P schemes adjust payments based on equity (socio-economic status of the target population or area) or based on structural quality is relevant for provider behaviour since both complicate the reward and reporting process for providers. Furthermore, adjustments based on socio-economic status can influence providers’ views on whether the playing field is level, or whether targets are achievable.

The final design feature we highlight is whether P4P schemes include safeguards for gaming (i.e. individuals manipulating P4P schemes to increase compensation). Such safeguards include verification or performance audits, and whether audits are associated with penalties or naming and shaming – which may tap into social norms about cheating [33].

### Systematic literature review

We carry out a review of studies on P4P schemes in LMIC to describe the designs of schemes and assess the completeness of reporting on incentive design within studies. To identify relevant P4P literature in LMIC we build on a search and screening strategy developed for a realist review on P4P by some of the authors of this paper [35]. As described in [35] to capture academic publications; Medline, Embase, and EconLit databases were searched. Additionally, to include relevant grey literature, Google Scholar, Emerald Insight, and websites of the World Bank, WHO, Cordaid, Norad, DfID, USAID and PEPFAR were searched. Two groups of search terms were used relating to P4P as well as LMICs – which are shown in Appendix A. The search period covered January 1, 1995 until 1st of January 2018. Reference lists of included studies were explored for additional relevant literature.

As is described in more detail in [35], the following inclusion criteria were used. First, studies needed to provide evidence on a health-related P4P intervention, targeting providers with financial incentives based on pre-specified performance targets. To discriminate P4P schemes from fee-for-service systems, only schemes that reward a sub-set of specified healthcare services were included. Second, the intervention had to be implemented in a LMIC, as defined by the World Bank [36]. Quantitative and empirical qualitative studies with any research design were included. The following exclusion criteria were applied. First, studies that assessed schemes targeting users or beneficiaries of healthcare services (i.e. demand-side incentives) as well as those describing fee-for-service system (targeting all, rather than a defined subset, of healthcare services) were excluded. Second, studies that discussed the potential implementation of P4P schemes not yet in place were excluded.

Using the full-text articles identified in [35] as a starting point, we applied three additional exclusion criteria for the purpose of this review. We excluded articles that provide no details on the P4P scheme in question – meaning that the scheme cannot be identified and it is not possible to verify whether the scheme is already described in another study. We excluded studies that discussed multiple P4P schemes, as well as literature reviews. We excluded studies published in a language other than English. Finally, papers that evaluated broader health financing policy packages, of which P4P was only one component – which was not explicitly described or focused on within the paper – were excluded.

**Fig. 2:**
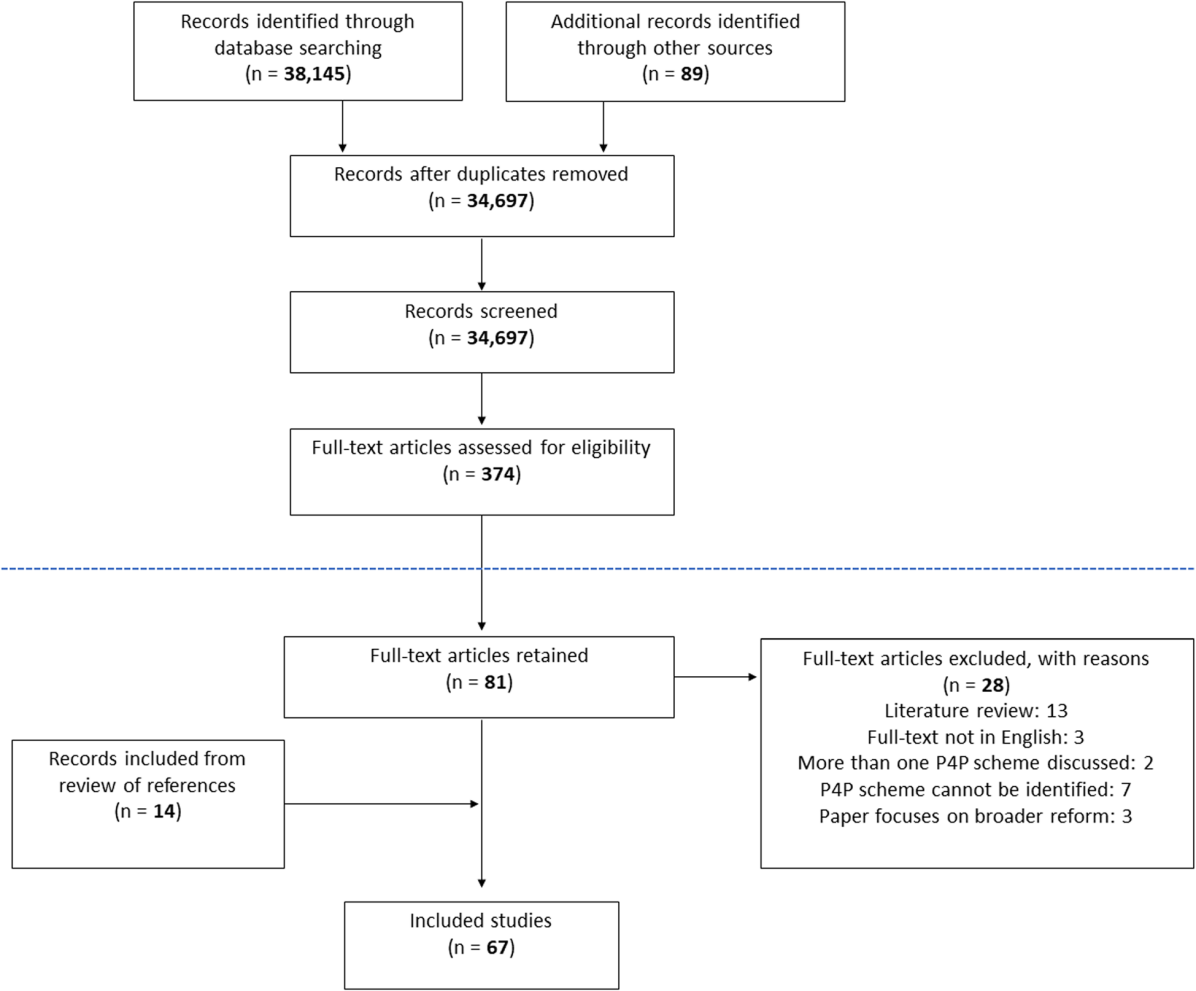
PRISMA flow chart of search strategy [37]. Elements in the diagram above the dotted line refer to work done for the realist review [35]. Elements below the dotted line refer to additional screening done for the purpose of this review

The search strategy identified 34,697 records and retained 81 articles after full-text screening based on the inclusion and exclusion criteria of the realist review [35]. As shown in Fig. 2, we excluded an additional 28 studies using the exclusion criteria set out above. We add a further 14 based on a review of references of included studies as well as one additional study identified through deliberation with researchers working on a Cochrane review on P4P in LMICs (updating a 2012 review [5]).

Data were extracted and recorded in Excel. First, we extracted basic information about studies (i.e. setting, study design, name of P4P scheme). Second, we extracted information on each of the design features shown in the typology. In case of multiple papers discussing the same scheme, we began by extracting data from the earliest publication, and where this did not provide information on all design elements of interest, more recent publications were subsequently reviewed. We only aimed to capture scheme design at a given point in time and did not take into account design changes that might have occurred over time – even when these were reported. We did not encounter situations in which publications on the same scheme (referring to the same time period) offered contradictory information on design. Finally, to obtain some information on the context in which schemes were implemented, we extracted information on: who implemented the scheme, who funded the scheme, whether external technical assistance was provided and whether any additional reforms were implemented alongside the P4P scheme.

To get an indication of the inter-rater reliability of the typology, a second reviewer extracted data for a random sample of 20% of identified schemes (i.e. 8 schemes). We find that Krippendorff’s alpha is 0.69, which is substantial. These results, while no more than illustrative, suggest that the typology was sufficiently simple to be applied in a reliable manner.

## Results

### Identified P4P schemes

**Fig. 3:**
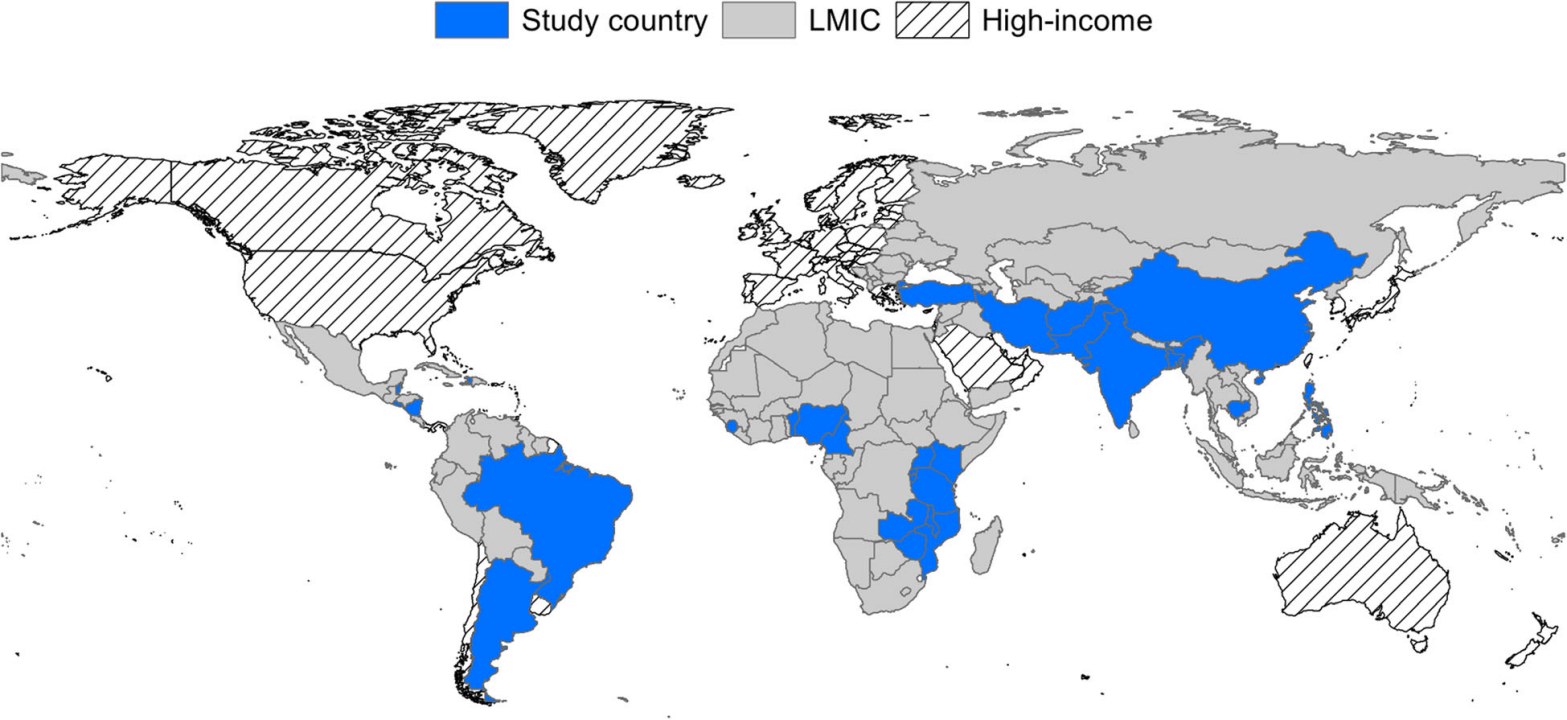
Map of countries included in the review. The figure was produced by the authors and is not taken from another source

The 67 studies identified in the review referred to 41 different P4P schemes, implemented in 29 LMICs (Fig. 3): Afghanistan (*n* = 1), Argentina (*n* = 4), Bangladesh (*n* = 2), Belize (*n* = 1), Benin (*n* = 2), Brazil (*n* = 1), Burundi (*n* = 2), Cambodia (*n* = 2), Cameroon (*n* = 1), China (*n* = 5), Democratic Republic of Congo (DRC) (*n* = 2), El Salvador (*n* = 1), Haiti (*n* = 1), India (*n* = 2), Iran (*n* = 2), Kenya (*n* = 1), Malawi (*n* = 1), Mozambique (*n* = 2), Nicaragua (*n* = 1), Nigeria (*n* = 1), Pakistan (*n* = 1), Philippines (*n* = 3), Rwanda (*n* = 12), Sierra Leone (*n* = 2), Tanzania (*n* = 8), Turkey (*n* = 1), Uganda (*n* = 1), Zambia (*n* = 2) and Zimbabwe (*n* = 1). The majority of the schemes reported on were in Africa (*n* = 20), followed by Asia (*n* = 12), Latin America (*n* = 6) and the Middle East (*n* = 3). Of the identified Schemes 54% were implemented in middle-income countries. In most countries (65%), only one P4P scheme was reported on. In some countries more than one scheme was reported on. For example, in Bangladesh, Burundi, Argentina, Zimbabwe, India, the Democratic Republic of the Congo, Tanzania, Rwanda and Cambodia two different P4P schemes were reported in the literature and in China four different schemes were reported.

The majority of the 41 schemes were implemented in primary care facilities (76%), with fewer being reported in hospitals (12%), at multiple levels of care (8%),^2^ in schools (2%), or with community health workers (2%). Schemes were primarily in public-sector facilities (60%) or in a mix of public and private facilities (33%).

Not all identified P4P schemes have received an equal amount of research attention. The majority of schemes are discussed in only one (76%) or two (14%) of the 67 included studies. However, schemes such as the *Plan Nacer* intervention in Argentina, the Philippine *Child Health Experiment*, a P4P pilot scheme in Pwandi Tanzania and Rwanda’s national P4P scheme were, respectively, the subject of three, three, seven and eleven identified studies. The full list of identified P4P schemes as well as the number of studies per scheme are shown in Appendix B.

### Incomplete reporting on design

The identified academic and grey literature on P4P in LMICs provided incomplete information relating to P4P scheme design, and there was variation in what was reported on across studies. The information gaps are particularly apparent when it comes to reporting who ultimately receives P4P payments, the attributes of the payment, the basis for payment as well as gaming safeguards.

**Fig. 4:**
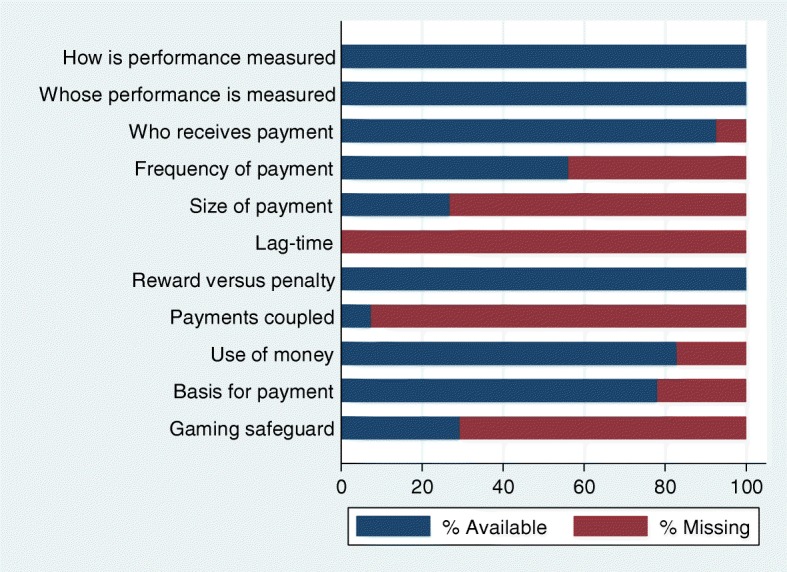
Missing information on design for 41 identified P4P schemes

As shown in Fig. 4, studies generally reported on how performance is measured, whose performance is measured and whether schemes provide rewards or penalties. The design features for which reporting was poorest was the time-lag between individuals reporting performance and receiving payments – which could not be retrieved for any of the identified schemes – as well as reporting on whether or not incentive payments are coupled with salary payments (available for 7% (3) of the 41 schemes). Part of the reason for this could be that these two features are not commonly monitored and are challenging to measure without primary data collection.

Design features that are arguably easier to capture but were nonetheless often omitted include: the average size of payments (missing for 73% (30) of studies) and whether there are safeguards for gaming (missing for 71% (29) of schemes), information on the frequency of the payment (missing for 44% (18) of schemes) and information on the basis for payment (missing for 22% (9) of schemes).

In addition, where design features were reported, this information was often difficult to find. More often than not, information on scheme design was not given in one section of the paper but often scattered across the paper, making it challenging for readers to get a good overview of how the P4P scheme was designed. An additional issue for P4P schemes reported on in more than one paper is that information on scheme design might only be available in one of the papers, and this is only rarely brought to readers’ attention through cross-referencing.

### Design of P4P schemes in LMICs

The review indicates that there is considerable heterogeneity in the way P4P schemes have been designed. Table 1 provides an overview of our main findings, which we describe below.

**Table 1:** Design of P4P schemes in LMICS (41 schemes in 29 LMICs)

Design features		Proportion of schemes [Number of schemes]
Measures of performance incentivised (*N* = 41) ^a^		
Healthcare visits		83% [34]
Quality of care (process)		66% [27]
Health outcomes		17% [7]
Quality of care (structural)		27% [11]
Management practices		22% [9]
Efficiency		5% [2]
Whose performance measured (*N* = 41) ^a^		
Individuals		26% [10]
Groups of health workers		3% [1]
Health facility		76% [31]
Health system managers		5% [2]
Who (ultimately) receives the payment (*N* = 38) ^a^		
Individuals		86% [32]
Groups of health workers		3% [1]
Health facility		46% [17]
Health system managers		10% [4]
Payment attributes		
(*N* = 23)	Frequency	
	Monthly or weekly	44% [10]
	Bi-monthly or quarterly	30% [7]
	Every 6 months	13% [3]
	Annual or one-off	13% [3]
(*N* = 11)	Median size	10% of monthly income
(*N* = 0)	Lag time	–
(*N* = 41)	Reward versus penalty	
	Rewards	98% [38]
	Penalties	2% [1]
(*N* = 3)	Coupled payments	
	Yes	67% [2]
	No	33% [1]
(*N* = 34)	Use of money	
	Staff income	56% [19]
	Operating budget	6% [2]
	Both	38% [13]
Basis for payment		
(*N* = 32)	Each action (e.g. visit)	
	Yes	72% [23]
	No	28% [9]
(*N* = 32)	Threshold target (single target)	
	Yes	28% [9]
	No	72% [23]
(*N* = 32)	Threshold target (multiple targets)	
	Yes	9% [3]
	No	91% [29]
(*N* = 41)	Type of ranking	
	Relative ranking (tournament)	5% [2]
	Own performance (absolute)	76% [31]
	Own performance (improvement)	19% [8]
(*N* = 41)	Payment adjustment	
	Equity	22% [9]
	Quality	19% [8]
	None reported	59% [24]
Gaming safeguards		
(*N* = 41)	Performance audit	
	Yes (without penalties reported)	61% [25]
	Yes (with penalties)	5% [2]
	None reported	34% [14]

Note: ^a^ = multiple options possible

#### What measure of performance is incentivised?

Basic information on which measure of performance was incentivised was provided for all identified P4P schemes. Healthcare visits and process quality of care were the most commonly incentivised performance measures within schemes in LMIC, in 83 and 66% of schemes respectively. For example, a P4P scheme in Afghanistan incentivised the number of ante-natal and post-natal consultations done [10] and a P4P scheme in Benin paid providers according to the number of curative consultations completed [38]. A P4P scheme in Kenya rewarded measures of process quality of care, including the proportion of patients correctly given antimalarial drugs (based on test results) [39]. Indicators of structural quality of care were rewarded in 11schemes – for instance, the availability of waste management and infection control equipment in a P4P scheme in Zimbabwe [40]. Management practices were incentivised in nine schemes – for example, maintenance of medical records was incentivised in a P4P scheme in Fengsan Township China [41]. Health outcome measures and efficiency were least commonly incentivised. Only seven schemes incentivised health outcome measures – for example, a scheme in India rewarded improvements in the nutritional status of children [15]. Only two schemes incentivised efficiency, for example, a hospital-based P4P scheme implemented at the national level in Turkey provided financial rewards for higher bed occupancy rates and shorter average length of stay [42].

Most P4P schemes (81%) rewarded multiple outcomes or activities – most commonly, the number of healthcare visits as well as measures of process quality of care. Few schemes rewarded only one activity. For instance, a P4P scheme in Zimbabwe focused on HIV prevention [43] tied payments exclusively to the number of male circumcisions and a P4P intervention in Chinese schools [44] only incentivised students’ haemoglobin level. Schemes rewarding multiple outcomes differed dramatically in their complexity. Some schemes rewarded just a handful of indicators, for instance a P4P scheme in Haut-Katang in the DRC rewarded seven indicators [45] or a P4P scheme in Haiti rewarded 14 indicators [46]. However, others are extremely complex, a good example is Brazil’s national P4P scheme (called PMAQ – National Program for Improving Primary Care Access and Quality), which rewarded over 600 different indicators [47]. Often studies only reported a sub-set of incentivised indicators (for instance, those that are being measured or assessed within the study) and do not make it clear which other indicators are incentivised by the programme. As a result, it can be difficult to ascertain the full set of activities that are incentivised by some schemes.

#### Who is incentivised?

Information on whose performance was measured was provided for all identified P4P schemes and information on who ultimately received the payment was available for 38 out of 41 schemes. Most identified P4P schemes (76%) measured the performance of health institutions such as primary healthcare facilities [46] or hospitals [48]. However, 86% of P4P schemes in LMICs paid financial bonuses to individual providers. Some schemes, such as a P4P scheme in Nigeria [49], distributed bonuses to healthcare providers of all cadres, whilst other schemes, such as a scheme in Ningxia, China [11] only paid doctors. It was not uncommon for schemes to measure the performance of one actor and provide payment to another actor. For instance, Brazil’s national P4P scheme measured the performance of primary healthcare facilities but paid municipal health managers [47].

#### How is performance incentivised?

There was considerable diversity in the way in which P4P schemes in LMICs designed the payment system, in terms of the payment attributes, the basis for payment and gaming safeguards.

In terms of payment attributes, information on the frequency with which P4P bonuses were paid was available for 23 of the included schemes. Across these studies, most schemes provided monthly payments, as was the case for instance in a scheme in China [44]. However, some schemes paid bonuses only once a year – such as the *Open Performance Review and Appraisal System* in Tanzania [50].

Some information on the level of payment per target was available for 23 schemes, however, information on the average payment size as a proportion of providers’ salary was only available for 11 of these. Across these studies, the median amount that providers earnt through P4P schemes was 10% of their monthly income. However, there was considerable variation across schemes; bonuses in a scheme in the Philippines amounted to 5% of providers’ monthly salary [51], compared to 38% of providers’ monthly salary for a scheme in Rwanda [9], one monthly salary for a scheme in Bangladesh [13] and twice the average monthly salary of health providers for a scheme in China [44]. Further information on the size of payments in different schemes is provided in Appendix C.

Even though the lag between when performance results are reported and when bonus payments are received is likely important for behaviour, this information was provided for none of the identified schemes.

Virtually all of the P4P schemes we identified provided financial rewards rather than penalties. Only one scheme, the *Iranian National Hospital Grading Programme* [48], included penalties as performance in the scheme was linked to how much hospitals can charge for patient days and hospitals with insufficient performance scores were closed.

Information on whether P4P payments are coupled with salary payments was available for only three identified schemes. Among these schemes, two reported payments taking place at the same time as salary payments – for instance a P4P scheme in Battagram district, Pakistan [52]. For a P4P scheme in Kisoro district, Uganda we inferred that payments were decoupled, as incentivised providers do not receive any base pay [53].

P4P schemes differ in the degree to which providers have autonomy about how additional funds can be used. In just over half (56% or 19) of the identified P4P schemes, incentives could exclusively be paid as a salary top-up to staff. This was for instance the case for a scheme in Chandigarh, India [15] or in the *Rural Mutual Health Care Programme* in Fengsan Township, China [41]. In 38% (13) of schemes P4P bonuses could be spent as a salary top up as well as the operating budget of healthcare facilities – to, for instance, purchase drugs and equipment. Examples of such schemes are the P4P scheme in Pwani Tanzania [54] or Sierra Leone’s national P4P scheme [55]. Notably, nine of the identified schemes specified how P4P bonuses had to be split between salary top-ups and facility running costs. For instance in the P4P scheme in Pwani Tanzania [54], up to a quarter of the bonuses could be used at the facility-level to purchase drugs and supplied or undertake minor renovations, with the remainder to be used for salary top ups. We only identified two schemes in which resources could exclusively be spent on facilities’ operational budget – a P4P scheme in Kenya’s Rift Valley [39] and the *Service Delivery Integration Programme* in Malawi [14].

Complete information on the basis for bonus payment was given for 32 of the identified schemes. Among these schemes, the majority (72%) provided financial rewards *per activity*. For example, a P4P scheme in Rwanda [56] offered a financial bonus for each client tested for HIV. The remaining schemes provided payments on the basis of a *threshold* being reached. Most (28%) used a single threshold. For instance, a scheme in Cambodia [57] provided payment as soon as 40% of deliveries in the catchment area were attended by a trained provider or when 75% of children in the catchment area were fully immunised. Few schemes (9%) used multiple thresholds. For example, a scheme in Argentina [58] paid providers an increasing amount depending on whether they screened over 17, 24% or 30% of patients for colorectal cancer.

All identified schemes provided some information on the type of performance ranking. The majority (76%) of P4P schemes in LMICs paid providers according to *absolute* performance. For example, a P4P scheme in Afghanistan rewarded the number of first ante-natal or post-natal care visits [10]. Some schemes (19%) rewarded health facilities for *improvements* in performance. For instance, some targets in a P4P scheme in Tanzania [16] gave rewards for an *increase* over time in the proportion of women in the catchment area using long-term contraceptives. Only two schemes (5%) used a *relative* rank or tournament design – in which facilities competed against one another for a given amount of money. For example, in a P4P scheme in China’s Ningxia province, healthcare centres that performed better than the average participating centre earnt larger bonuses [11].

The majority of P4P schemes in LMICs did not incorporate equity into the payment calculation to allow, for example, providers to earn larger bonuses if they reached targets for disadvantaged groups or households living in disadvantaged areas. Of the identified schemes, nine (22%) included some form of equity adjustment. For instance, in a P4P scheme in Cambodia [57], healthcare workers earnt additional bonuses for increases in the healthcare use of people classified as being in a low “socio-economic stratum” (p.199). Most schemes do not adjust the overall amount earned based on quality of care. Only 19% of identified schemes – many of which were in sub-Saharan Africa, for instance in Burundi [55], weigh provider performance using a quality index or score to determine the amount of money earned.

Some form of safeguarding against gaming was reported for almost all schemes. In 61% (25) of schemes, facilities were audited to determine whether actual and reported performance were consistent. Studies rarely provided information on who conducted the audits, and whether they were performed by independent surveyors, which have no conflict of interest. In some settings, for instance in a P4P scheme in Pwani Tanzania [54], audits were carried out by health system manages, who are also incentivised based on the performance of facilities. For only two schemes (5%) studies reported that audits were connected to penalties for misreporting.

### Implementation, funding and complementary reforms

Table 2 provides an overview of implementation, funding and complementary reforms for identified schemes. Information on who implemented the scheme was provided for 33 of the 41 identified schemes. Most identified P4P schemes were implemented by non-governmental or international organisation (58%) or by regional or national governments (24%). The majority of P4P schemes in LMICs (68%) were implemented with some form of external technical assistance by international NGOs or international organisations – as was the case in Haiti [46] Afghanistan [10] and Bangladesh [59]. All included schemes implemented in low-income settings were implemented with some form of external technical assistance, compared to 50% of schemes in middle-income settings. Information on who funded the scheme was provided for 39 of the 41 identified schemes. Most schemes were funded by governmental bodies (national or regional) (43%), non-governmental or international organisations (including bilateral or multi-lateral donors) (28%) or a collaboration between non-governmental or international organisations and governmental bodies (18%).

**Table 2:** Funding, implementation and complementary reforms

Funding, implementation and complementary reforms		Proportion of schemes [Number of schemes]
(*N* = 33)	Main implementer	
	Government body	24% [8]
	Health facility	3% [1]
	International or non-governmental organisation & government body	12% [4]
	International or non-governmental organisation	58% [19]
	Researchers	3% [1]
(*N* = 39)	Main funder	
	Government body	43% [17]
	Health facility	3% [1]
	International or non-governmental organisation & government body	18% [7]
	International or non-governmental organisation	28% [11]
	Researchers	8% [3]
(*N* = 41)	External technical assistance	68% [28]
(*N* = 41)	Complementary reforms	51% [21]

Some differences in scheme design appear to be influenced by the organisation funding and implementing the scheme. For instance, among the ten schemes funded by the World Bank, many measure the performance of facilities in terms of service volumes, provide bonuses to healthcare providers and include design features such as a quality index^3^ to weight performance – as was the case in schemes in Benin [38], Pakistan [52] or Rwanda [9].

Notably, half of the identified P4P schemes were implemented at a similar time as other health systems reforms (such as increased supervision, training activities, roll-out of health insurance, changes in user-fees), which substantially limits the scope to robustly evaluate their impacts. For instance, in Burundi, a P4P scheme was implemented at a similar time as an intervention that removed user-fees for under-five year olds [60].^4^

## Discussion

This study examines the design of P4P schemes in healthcare in LMICs and includes 67 studies published in the academic and grey literature. The review identified 41 different P4P schemes implemented in 29 LMICs. First, we found that while the literature often refers to P4P as if it were a uniform intervention, there is in fact substantial heterogeneity in the design of P4P schemes in LMICs. This is perhaps not surprising, given the number of design choices that need to be made when designing a P4P scheme – which is common for complex health systems interventions that are intended to be flexible to meet local needs and priorities. We are able to pinpoint how and to what degree P4P schemes differ in terms of design – which substantiates previous, more general, remarks about differences in scheme design from high-income settings [21, 22] and LMICs [5]. Results suggest that schemes differ substantially in terms of which performance measures are incentivised, whose performance is measured, who receives payments, the payment attributes (frequency, size, etc.), the basis for payment (rewarding each action or a threshold of performance, the type of ranking used, etc.) and the existence of gaming safeguards. This evidence strongly supports the idea that there does not appear to be a standard blueprint for P4P scheme design being used in LMICs. This variation may well reflect the fact that P4P schemes have been tailored to meet certain policy objectives and respond to different conditions on the ground. At the same time, it is plausible that some of the heterogeneity in effectiveness reported by different studies can be attributed to differences in scheme design (alongside other factors such as implementation fidelity and context). The theory we draw on to develop our typology may be relevant for policymakers when designing P4P schemes, as it provides some guidance on the advantages and drawbacks of different design choices. In addition to these key differences in scheme design, which a priori affect how individuals and groups respond to incentives, there are also several shortcomings in the way scheme design is reported on in the identified studies. There is variation in what is reported across studies, and important design information is often missing from studies, for example information on who receives P4P payments, payment attributes (frequency, size, lag-time, coupling of payments with providers’ salary), how money can be used and whether there are gaming safeguards. In addition, even when such information could be identified, it was often difficult to retrieve as it was spread across several sections of the paper or contained in previous publications that were not referenced directly.

Given the substantial heterogeneity in terms of P4P scheme design, we recommend that authors of future studies pay more attention to describing the design of schemes they are evaluating. Current reviews of P4P are limited in what they can say about the effectiveness of P4P schemes without capturing incentive design, as this is key to understanding which types of schemes work and which do not in different settings. Indeed, the heterogeneous effects of P4P schemes observed in LMICs, could at least partially be attributed to differences in scheme design. Therefore, reporting information on scheme design is a first step towards understanding which design features are consequential for outcomes. Such analyses would be highly relevant for policymakers seeking to design incentives that work. In this study, we provide a simple typology that can be used as a starting point for reporting P4P scheme design in future studies in LMICs (see Table 1).

This review is limited in four main respects. First, we only examine academic and grey literature to get an overview of how schemes are designed in LMICs. An alternative approach would have been to directly seek out policy documents from ministries of health or NGOs implementing P4P schemes in order to retrieve more detailed information on design. The main reason why we did not adopt this strategy was that we were specifically interested in examining how P4P schemes are being reported on in the academic and research-focused grey literature.

Second, to identify relevant papers we rely on a search strategy developed for a closely related review conducted by some of the authors. Given that the two reviews had slightly different objectives (one focusing on the design of P4P schemes, the other focusing on how and in what settings P4P schemes affect outcomes) – it is possible that some relevant papers could have been screened out. To ensure that relevant papers were captured, we screened the references of identified studies. We also compared the list of schemes we identified to those included in a soon to be published Cochrane review on P4P in LMICs (updating a 2012 review [5]). We are therefore reasonably confident that all relevant P4P schemes discussed in the academic and research-focused grey literature were captured in this study.

Third, we did not address the important question of whether scheme design affects outcomes as this would have been difficult given the available data. To begin with, the studies use very different outcomes, varying from childhood stunting to patient satisfaction or provider motivation, limiting our ability to compare differences in effect sizes across studies. Secondly, the degree of heterogeneity in scheme design (considering combinations of design features) is very large relative to the number of observations. It is possible that in the future such an analysis could be feasible – assuming that reporting on incentive design improves and the number of published studies increases. However, at the moment it would not be possible to quantitatively examine the link between design and outcomes.

Furthermore, we only describe the design of P4P schemes based on the dimensions of our typology. As a consequence, we only record selected design features. This leaves open the possibility that we did not record information on potentially relevant design elements. After we developed our typology, an article providing a reporting framework and typology for categorising P4P schemes was published [25]. As detailed in Appendix D, the typology developed here includes all of the elements identified in [25]. We also report more details on identified design features and include several additional design features pertinent to P4P schemes in LMICs, specifically: whose performance is measured, the frequency of payments, how money can be used, whether there is payment adjustment for equity or quality as well as information on gaming safeguards – which are not captured by [25]. This supports the completeness of our typology.

Finally, as this review focuses on incentive design, we do not provide evidence on related contextual factors that likely affect performance, for example the degree to which P4P schemes promote autonomy. Previous studies indicate that if employees feel that they have sufficient autonomy, incentives are less likely to be viewed as an attempt to control their behaviour but rather as an affirmation of their competence – which can increase intrinsic motivation and improve performance [61, 62]. On the one hand, many P4P schemes implemented in the African region, were often accompanied by reforms aimed at financial decentralisation (e.g. introducing facility bank accounts), giving health facilities more autonomy in how they operate [35]. On the other hand, P4P schemes are sometimes characterised as reducing autonomy, by incentivising them towards specified actions at the expense of autonomy in clinical decision making [35]. Although we did not explicitly examine the degree of provider autonomy in different P4P schemes, there does appear to be variation across schemes in this regard. For instance, we examine how money raised through P4P bonuses can be used. We find that some schemes only allow bonus payments to be used as salary top-ups [15, 41], whilst others allow bonuses to be spent on staff as well as facility operations [54, 55] – which may allow facilities to determine how to allocate bonuses.

We have three main recommendations for future research. First, we encourage authors to improve the consistency and completeness of reporting on P4P scheme design, given the considerable heterogeneity in designs between settings and the potentially important implications for understanding scheme effectiveness. We suggest using the typology laid out in this paper as a starting point. Second, in order for a review on the effect of P4P scheme design on outcomes to be viable in the future, studies evaluating P4P schemes should make an attempt to report potential effects in a more standardised way – for instance, in terms of standard deviation or percentage point changes – and ideally examine a more comparable set of outcomes. Finally, we encourage authors to examine how differences in design affect outcomes within a country. It is not uncommon for P4P schemes to change design over time, or between areas, and authors should make an attempt at exploiting such variation.

## Conclusions

We find that there is substantial heterogeneity in the design of P4P schemes in LMICs and pinpoint precisely how scheme design varies across settings. Our results also underline that incentive design is not adequately being reported on in the literature. We encourage authors to make a greater effort to report information on P4P scheme design in the future and suggest using the typology laid out in this paper as a starting point.

## Supplementary information

**Additional file 1.Appendix A**. – Search strategy. **Appendix B**. – Overview of P4P schemes included in the review. **Appendix C**. – Size of P4P incentives. **Appendix D**. – Identified design features. [63–96]

## Data Availability

The datasets used and/or analysed during the current study are available from the corresponding author on reasonable request.

## Abbreviations

Cordaid: Catholic Organization for Relief and Development Aid
DfID: Department for International Development (UK)
LMIC: Low and middle-income country
NGO: Non-governmental organisation
Norad: Norwegian Agency for Development Cooperation
P4P: Pay for performance
PEPFAR: President’s Emergency Plan for AIDS Relief
PRISMA: Preferred Reporting Items for Systematic Reviews and Meta-Analyses
USAID: United States Agency for International Development
WHO: World Health Organization

## Footnotes

1 These design variations are common with many complex health systems interventions, which are intended to be flexible and give autonomy to implementers to tailor programmes to the local context.

2 One P4P scheme was implemented in hospitals and secondary care clinics and two were implemented in hospitals and primary care facilities.

3 Often referring to structural quality of care (access to drugs and equipment, infrastructure) in the facility.

4 They note that “PBF pilot projects started in Burundi in 2006, at the same time when a national policy removed user fees for pregnant women and children below 5 years old”.
