# Supplementary material for: How are pay-for-performance schemes in healthcare designed in low- and middle-income countries? Typology and systematic literature review
Source: BMC Health Serv Res. 2020 Apr 7;20:291. doi: 10.1186/s12913-020-05075-y (PMC7137308; doi:10.1186/s12913-020-05075-y)
Supplement: Supplementary file 1 — Additional file 1.Appendix A. – Search strategy. Appendix B. – Overview of P4P schemes included in the review. Appendix C. – Size of P4P incentives. Appendix D. – Identified design features. [63–96] [file 12913_2020_5075_MOESM1_ESM.docx]

# Appendix A – Search strategy

Master search strategy adapted for peer-reviewed and grey literature databases in the realist review. Search terms were first developed for Medline and then adapted to the other databases.

| Topic | Search syntax |
| --- | --- |
| LMICs | Developing Countries/ |
|  | Medically Underserved Area/ |
|  | Medically Underserved Area/ |
|  | exp "Africa South of the Sahara"/ |
|  | exp Asia/ |
|  | exp South America/ |
|  | exp Latin America/ or exp Central America/ |
|  | (Africa or Asia or South America or Latin America or Central America).tw. |
|  | (Albania or Algeria or Angola or Armenia or Azerbaijan or Belarus or Bhutan or Bolivia or "Bosnia and |
|  |  |
|  | Herzegovina" or Cameroon or China or Colombia or Congo or Cuba or Djibouti or Dominican Republic or Ecuador |
|  | or Egypt or El Salvador or Fiji or "Georgia (Republic)" or Guam or Guatemala or Guyana or Honduras or Indian |
|  | Ocean Islands or Indonesia or Iran or Iraq or Jamaica or Jordan or Lesotho or "Macedonia (Republic)" or Marshall |
|  | Islands or Micronesia or Middle East or Moldova or Morocco or Namibia or Nicaragua or Paraguay or Peru or |
|  | Philippines or Samoa or Sri Lanka or Suriname or Swaziland or Syria or Thailand or Tonga or Tunisia or |
|  | Turkmenistan or Ukraine or Vanuatu).sh,tw. or Bosnia.tw. or Cape Verde.tw. or Gaza.tw. or Georgia.tw. or |
|  | Kiribati.tw. or Macedonia.tw. or Maldives.tw. or Marshall Islands.tw. or Palestine.tw. or Syrian Arab Republic.tw. |
|  | or West Bank.tw. |
|  | (American Samoa or Argentina or Belize or Botswana or Brazil or Bulgaria or Chile or Comoros or Costa Rica or  Croatia or Dominica or Equatorial Guinea or Gabon or Grenada or Hungary or Kazakhstan or Latvia or Lebanon or  Libya or Lithuania or Malaysia or Mauritius or Mexico or Micronesia or Montenegro or Oman or Palau or Panama  or Poland or Romania or Russia or Seychelles or Slovakia or South Africa or "Saint Kitts and Nevis" or Saint Lucia  or "Saint Vincent and the Grenadines" or Turkey or Uruguay or Venezuela or Yugoslavia).sh,tw. or Guinea.tw. or  Libia.tw. or libyan.tw. or Mayotte.tw. or Northern Mariana Islands.tw. or Russian Federation.tw. or Samoa.tw. or  Serbia.tw. or Slovak Republic.tw. or "St Kitts and Nevis".tw. or St Lucia.tw. or "St Vincent and the  Grenadines".tw. |
|  | (Afghanistan or Bangladesh or Benin or Burkina Faso or Burundi or Cambodia or Central African Republic or Chad  or Comoros or "Democratic Republic of the Congo" or Cote d'Ivoire or Eritrea or Ethiopia or Gambia or Ghana or  Guinea or Guinea-Bissau or Haiti or India or Kenya or Korea or Kyrgyzstan or Laos or Liberia or Madagascar or  Malawi or Mali or Mauritania or Melanesia or Mongolia or Mozambique or Myanmar or Nepal or Niger or  Nigeria or Pakistan or Papua New Guinea or Rwanda or Senegal or Sierra Leone or Somalia or Sudan or Tajikistan  or Tanzania or East Timor or Togo or Uganda or Uzbekistan or Vietnam or Yemen or Zambia or Zimbabwe).sh,tw.  or Burma.tw. or Congo.tw. or Kyrgyz.tw. or Lao.tw. or North Korea.tw. or Salomon Islands.tw. or Sao Tome.tw. or  Timor.tw. or Viet Nam.tw. |
|  | ((developing or less$ developed or third world or under developed or middle income or low income or  underserved or under served or deprived or poor$) adj (count$ or nation? or state? or population?)).tw. |
|  | (lmic or lmics).tw. |
| P4P | Employee Incentive Plans/ and (financ$ or pay$ or incentive? or initiative? or bonus$).tw. |
|  | Physician Incentive Plans/ and (financ$ or pay$ or incentive? or initiative? or bonus$).tw. |
|  | Reimbursement, Incentive/ and (financ$ or pay$ or incentive? or initiative? or bonus$).tw. |
|  | Fee-for-Service Plans/ and (financ$ or pay$ or incentive? or initiative? or bonus$).tw. |
|  | Contract Services/ and (financ$ or pay$ or incentive? or initiative? or bonus$).tw. |
|  | (conditional adj3 (pay$ or transfer?)).tw. |
|  | (pay$ adj2 performance).tw. |
|  | (Results adj3 financing).tw. |
|  | (Performance adj3 financing).tw. |
|  | (Performance adj3 scheme*).tw. |
|  | (Results adj3 incentive*).tw. |
|  | (Performance adj3 contracting).tw. |
|  | (Results adj3 contracting).tw. |
|  | "Fees and Charges"/ and (fee? or charge? or pay$).tw. |
|  | Fees, Dental/ and (fee? or charge? or pay$).tw. |
|  | Fees, Medical/ and (fee? or charge? or pay$).tw. |
|  | Fees, Pharmaceutical/ and (fee? or charge? or pay$).tw. |
|  | Prescription Fees/ and (fee? or charge? or pay$).tw. |
|  | Hospital Charges/ and (fee? or charge? or pay$).tw. |
|  | Capitation Fee/ and (fee? or charge? or pay$).tw. |
|  | Fee-for-Service Plans/ and (fee? or charge? or pay$).tw. |
|  | "Cost Sharing"/ and (fee? or charge? or pay$).tw. |
|  | "Cost Sharing"/ and (fee? or charge? or pay$).tw. |
|  | Outsourced Services/ and (fee? or charge? or pay$).tw. |
|  | Prepaid Health Plans/ and (fee? or charge? or pay$).tw. |
|  | Prospective Payment System/ and (fee? or charge? or pay$).tw. |
|  | Insurance, Health/ and (fee? or charge? or pay$).tw. |
|  | ((medical or dental or pharmac$ or dispensing or drug or drugs or medicament? or medicine? or prescript$ or |
|  | consultation? or treatment? or registration? or hospital? or care) adj2 (fee? or charge?)).tw. |
|  | ((user? or patient? or outpatient? or inpatient?) adj2 (fee? or charge? or pay$)).tw. |
|  | ((pay$ or cash or money or monetary or economic or financial) adj2 incentive?).tw. |
|  | (Conditional adj3 cash transfers).tw. |
|  | Cash transfer*.tw. |

# Appendix B – Overview of P4P schemes included in the review

| **Country** | **Programme name (name assigned during review = ^A^)** | **Identified studies** |
| --- | --- | --- |
| Afghanistan | P4P Afghanistan ^A^ | [10] |
| Argentina | P4P Argentina hospital-based ^A^ | [58] |
| Argentina | Plan Nacer Argentina | [61]–[63] |
| Bangladesh | P4P Bangladesh (small-scale) ^A^ | [13] |
| Bangladesh | P4P Bangladesh (Zakiganj and Kanaighat) ^A^ | [59] |
| Belize | P4P Belize ^A^ | [64] |
| Benin | P4P Benin ^A^ | [38], [65] |
| Brazil | PMAQ (Program for Improving Primary Care Access and Quality) | [47] |
| Burundi | P4P Burundi I ^A^ | [60] |
| Burundi | P4P Burundi II ^A^ | [66] |
| Cambodia | P4P Cambodia I ^A^ | [57] |
| Cambodia | P4P Cambodia II ^A^ | [67] |
| Cameroon | P4P Cameroon ^A^ | [68] |
| China | P4P China anaemia reduction ^A^ | [44] |
| China | P4P China (Ningxia) ^A^ | [11], [69] |
| China | P4P China (Shandong) ^A^ | [70] |
| China | Rural Mutual Health Care programme Fengsan Township | [41] |
| DRC | P4P DRC (South Kivu) ^A^ | [71] |
| DRC | DRC Haut-Katanga P4P | [45] |
| El Salvador | Salud Mesoamerica Initiative | [72] |
| Haiti | P4P Haiti (Santé pour le développement et la stabilité d’Hait) | [46] |
| India | P4P India (input and output incentives) ^A^ | [73] |
| India | P4P India Chandigarh (ICDS) | [15] |
| Iran | Iranian national hospital grading programme | [48], [74] |
| Kenya | P4P Kenya Rift Valley ^A^ | [39] |
| Malawi | Malawi Service Delivery Integration programme | [14] |
| Mozambique | P4P Mozambique ^A^ | [75], [76] |
| Nicaragua | Red de Protección Social | [77] |
| Nigeria | P4P Nigeria ^A^ | [49] |
| Pakistan | P4P Pakistan ^A^ | [52] |
| Philippines | Phillipine Child Health Experiment | [51][78][79] |
| Rwanda | P4P Rwanda ^A^ | [9], [56], [71], [80]–[88] |
| Rwanda | P4P Rwanda (Kabutare district) ^A^ | [89] |
| Sierra Leone | P4P Sierra Leone ^A^ | [55], [90] |
| Tanzania | Open Performance Review and Appraisal System | [50] |
| Turkey | P4P Turkey ^A^ | [42] |
| Tanzania | P4P Tanzania (Pwani) ^A^ | [16], [42], [54], [91]–[94] |
| Uganda | P4P Uganda (Kisoro) ^A^ | [53] |
| Zambia | P4P Zambia ^A^ | [95], [96] |
| Zimbabwe | P4P Zimbabwe ^A^ | [40] |
| Zimbabwe | P4P Zimbabwe voluntary male circumcision ^A^ | [43] |

# Appendix C – Size of P4P incentives

To calculate the size of the incentive in terms of a proportion of providers’ monthly salary, information from the 11 schemes shown below was used.

| **Scheme** | **Size of incentive** |
| --- | --- |
| P4P Bangladesh | 1 month of salary |
| Tanzania Open Performance Review and Appraisal System | 10% of monthly salary |
| P4P Tanzania (Pwani) | 10% of monthly salary |
| Rural Mutual Health Care programme Fengsan Township | 13% of monthly salary |
| P4P Zambia | 17% of monthly salary |
| Philippine child health experiment | 5% of monthly salary |
| P4P Rwanda | 38% of monthly salary |
| P4P Afghanistan (2010-2012) | 6-11% of monthly salary |
| P4P China (Shandong) | 10% of monthly salary |
| P4P Sierra Leone | 9% of monthly salary |
| P4P Anaemia china | Two months of salary (3000 Yuan) |

A further five schemes provided some information on the size of the incentive but did not comment on the size of payments as a proportion of providers’ salary.

| **Scheme** | **Size of incentive** |
| --- | --- |
| P4P China (Ningxia) | 12000 RMB per year |
| P4P Zimbabwe (VMC) | 25 USD per circumcision |
| P4P Bangladesh (Zakiganj and Kanaighat) | 41 USD per person |
| P4P Nicaragua (Red Protection social) | 9.3 USD per person |
| P4P Burundi I | Salaries before P4P = 75 USD, salaries during P4P = 262 USD |

# Appendix D – Identified design features

The table below compares the typology developed here to the one developed by Ogundeji, Sheldon and Maynard [25]. Ogundeji, Sheldon and Maynard capture whether schemes provide non-monetary incentives (such as gifts or material things) – a design feature not included here, as studies providing non-monetary incentives are not included.

The typology developed here is more detailed than the one developed by Ogundeji, Sheldon and Maynard. We capture whose performance is measured in P4P schemes, the frequency of payments, how money can be used, whether there is payment adjustment for equity of quality as well as information on gaming safeguards, which are not captured by [25].

| **Identified design features** | | **Typology by Ogundeji, Sheldon and Maynard** [25] |
| --- | --- | --- |
| Measures of performance incentivised | | Domain of performance measured |
|  | Healthcare visits | *Within clinicians’ control* |
|  | Quality of care (process) | *Out of clinicians’ control* |
|  | Quality of care (outcome) |  |
|  | Quality of care (structural) |  |
|  | Management quality |  |
|  | Cost (efficiency) |  |
| Whose performance measured | |  |
|  | Individuals |  |
|  | Groups of health workers |  |
|  | Health facility |  |
|  | Health system managers |  |
| Who (ultimately) receives the payment | | Who received incentive |
|  | Individuals | *Individuals* |
|  | Groups of health workers | *Groups* |
|  | Health facility |  |
|  | Health system managers |  |
| Payment attributes | |  |
|  | Frequency |  |
|  | Monthly or weekly |  |
|  | Bi-monthly or quarterly |  |
|  | Every 6 months |  |
|  | Annual or one-off |  |
|  | Average size | Size of incentive |
|  |  | *Large* |
|  |  | *Medium* |
|  |  | *Small* |
|  | Lag time | Time-lag |
|  |  | *Short* |
|  |  | *Long* |
|  | Reward versus penalty | Type of incentive |
|  | *Rewards* | *Bonus* |
|  | *Penalties* | *Fine* |
|  | Coupled payments | Method of payment |
|  | Yes | *Coupled* |
|  | No | *Decoupled* |
|  | Use of money |  |
|  | *Staff income* |  |
|  | *Operating budget* |  |
|  | *Both* |  |
| Basis for payment | |  |
|  | Each action (e.g. visit) | Payment mechanism |
|  | *Yes* | *Increase in performance* |
|  | *No* |  |
|  | Threshold target (single target) |  |
|  | *Yes* |  |
|  | *No* |  |
|  | Threshold target (multiple targets) | Payment mechanism |
|  | *Yes* | *Threshold* |
|  | *No* |  |
|  | Type of ranking | Payment scale |
|  | *Relative ranking (tournament)* | *Relative* |
|  | *Own performance (absolute)* | *Absolut* |
|  | *Own performance (improvement)* |  |
|  | Payment adjustment |  |
|  | *Equity* |  |
|  | *Quality* |  |
|  | *None reported* |  |
| Gaming safeguards | |  |
|  | Performance audit |  |
|  | *Yes (without penalties reported)* |  |
|  | *Yes (with penalties)* |  |
|  | *None reported* |  |
